# Characterization of Exopolysaccharide Produced by* Streptococcus thermophilus* CC30

**DOI:** 10.1155/2017/4201809

**Published:** 2017-07-26

**Authors:** Sri Lakshmi Ramya Krishna Kanamarlapudi, Sudhamani Muddada

**Affiliations:** Department of Biotechnology, KL University, Green Fields, Vaddeswaram, Guntur 522502, India

## Abstract

An exopolysaccharide (EPS) producing strain CC30 was isolated from raw milk and identified as* Streptococcus thermophilus* with morphological and 16S sequencing analysis. The strain was shown to produce 1.95 g/L of EPS when grown in skim milk lactose medium at 30°C by increasing the viscosity of the medium. The EPS was isolated and purified, and it was shown to consist of glucose and galactose in 1 : 1 ratio, with molecular weights ranging from 58 to 180 kDa. FTIR spectroscopy indicated the EPS to have amide, hydroxyl, and carboxyl groups. Under Atomic Force Microscopy, EPS showed spike-like lumps of EPS. Scanning Electron Microscopy (SEM) studies showed that it had irregular lumps with a coarse surface. The EPS displayed pseudoplastic nature. Thermogravimetric analysis (TGA) reported a degradation temperature of 110.84°C. The purified EPS exhibited reducing activity, hydrogen peroxide radical scavenging activity, and emulsification activity. The results of the present study indicated that EPS producing* Streptococcus thermophilus* could serve as a promising candidate for further exploitation in food industry.

## 1. Introduction

Many types of polysaccharides produced by algae, plants, and bacteria are most commonly used as texturizers, emulsifying agents, or viscosifiers in the food additives and to increase the consistency and texture of the fermented milk. The use of these polysaccharides might be attractive for the food industry when excreted during the manufacture of food and constitute a new generation of food thickeners [1]. Hence, during the last decade there was an increasing attention in the production of exopolysaccharide (EPS) by the lactic acid bacteria (LAB) which have been shown to play an important role in the prevention of whey separation (syneresis), during manufacturing of yoghurt. Also, because of their GRAS (Generally Regarded as Safe) status and rheological properties, the use in food fermentation could lead to the production of safe and natural products with improved stability. EPS are also reported to have therapeutic properties, such as antitumour, antioxidant, cholesterol lowering, antibiofilm, immunomodulatory, and immunostimulatory activities [2].

A variety of EPS can be obtained by exploiting the diversity of lactic acid bacteria. Among LAB strains* Streptococcus thermophilus* is known for its probiotic properties and general use as starter culture in yoghurt preparation. Based upon the strain, culture conditions, and media composition, the structure, composition, and yield of the EPS vary.

In the present study, the EPS produced from a strain of* S. thermophilus* CC30 was characterized by Gas Chromatography-Mass Spectrometry (GC-MS), Scanning Electron Microscopy (SEM), Atomic Force Microscopy (AFM), Thermogravimetric Analysis (TGA), and rheology for its potential application in the food.

## 2. Materials and Methods

### 2.1. Media Preparation and Culture Conditions

The skim milk lactose agar medium containing 11% skim milk, 0.35% yeast extract, 1% lactose, and 1.5% agar was used for screening of lactic acid bacteria strains. The strains were propagated in 11% skim milk for the production of exopolysaccharide. MRS (Deman, Rogosa, and Sharpe) medium was used as a growth medium for lactic acid bacteria. The cultures were propagated by incubation for 24 to 48 hrs at 30°C.

### 2.2. Microorganisms Used

Exopolysaccharide producing lactic acid bacterial strains were isolated from raw milk samples obtained from different milk collection centers in Guntur, Andhra Pradesh. Different dilutions of milk samples were spread on skim milk lactose [2] plates and incubated at 30°C for 48 hrs. The colonies were identified by Gram's staining. Gram-positive and catalase negative colonies were further propagated in 11% skim milk for exopolysaccharide production. Among the screened isolates, the strain designated CC30 showed viscous curdling and ropiness in skim milk. The strain was preserved by storing in MRS lactose broth containing 50% glycerol at −80°C.

### 2.3. Identification of Microorganisms

The sequence of 16S rRNA gene was performed at Macrogen Inc., (South Korea). The gene sequence was analyzed by BLAST and phylogenetic tree was constructed using ClustalW2.

### 2.4. Production and Isolation of EPS

The exopolysaccharide was produced by inoculating the strain, CC30, in 11% skim milk and incubating for 24 hrs at 30°C. Briefly, 100 mL of fermented milk was mixed with 12% trichloroacetic acid and incubated for 24 hrs. Bacterial culture and proteins were separated from the supernatant by centrifuging at 7000 rpm at 4°C for 20 min. EPS is precipitated from the supernatant by mixing with three volumes of cold absolute ethanol and incubated at 4°C for 48 hrs. The precipitated EPS was then separated by centrifugation (7000 rpm at 4°C for 20 min). The obtained EPS was dissolved in double distilled water and dialyzed at 4°C by using Centrikon (Pall, Nanosep centrifugal devices) with the molecular weight cut-off of 8–10 kDa. The obtained EPS fraction was freeze-dried and then used for further characterization studies.

### 2.5. Chemical Analysis of EPS

Protein content was determined by the method of Lowry et al., with bovine serum albumin as standard [3]. The total amount of carbohydrates present in the polysaccharide was determined by the phenol-sulfuric acid method as described by Dubois et al., using glucose as standard [4]. The nucleic acid content was determined by diphenylamine method, using DNA as standard [5].

### 2.6. Fourier Transform Infrared (FTIR) Analysis of EPS

Fourier Transform Infrared spectrum for the polysaccharide sample was acquired in transmittance mode with Thermo Nicolet, Avatar 370 spectrometer to analyze different functional groups. Compressed discs of 3 mm diameter were prepared by mixing with 2 mg of lyophilized EPS with 200 mg of KBr, and the spectrum was corrected for KBr background. The pellets were then scanned in the range of 4000–500 cm^−1^ with a resolution 4 cm^−1^ and using 32 scans.

### 2.7. Molecular Weight Estimation of EPS

The molecular weight of the polysaccharide was determined by size exclusion high performance liquid chromatography (SE-HPLC) of Agilent 1100 series equipped with a ZORBAX GF-250 column (9.4*∗*250 mm 4 microns) calibrated with 50 Mm Tris, 100 mM KCl (pH 8) buffer. Purified EPS was injected and was eluted with the same buffer at a flow rate of 1 mL/min. The linear regression was calibrated with several dextran standards (500 kDa, 100 kDa, 70 kDa, 40 kDa, and 6 kDa).

### 2.8. Monosaccharide Composition of EPS

To determine the monosaccharide composition of CC30 EPS, 5 mg of purified EPS was hydrolyzed with 2 mL of 2 M trifluoroacetic acid (TFA) at 120°C for 2 hrs. The hydrolysates were then reduced with potassium borohydride (KBH_4_) dissolved in ammonium hydroxide (NH_4_OH) and were subjected to N-acetylation using acetic anhydride (CH_3_CO)_2_O. The derivative products were used for determination of the monosaccharide composition by Gas Chromatography. Sample of 0.5 *μ*L was injected into the GCMS using autosampler. GC was performed on a Perkin Elmer Clarus SQ8 GCMS equipped with the autosampler and an RTX-5MS column (30 m length *∗* 0.32 mm inner diameter *∗* 0.25 *μ*m film thickness). Helium was used as the carrier gas with 1 mL/min flow rate. The chromatographic conditions used are as follows: the initial column temperature was held at 40°C for 1.5 min, increased at a rate of 40°C/min to 130°C, and then subsequently increased at 8°C/min to 290°C, where it was held at 290°C for 5 min. The comparison was made with standard glucose, galactose, rhamnose, arabinose, mannose, fucose, N-acetyl glucosamine, and N-acetyl galactosamine for sugar identification.

### 2.9. Rheological Analysis of Aqueous EPS Solution

The viscosity of the aqueous purified polysaccharide solution was measured using AR 2000 Visco-Rheometer. The viscosity measurements were performed at 25°C with increasing shear rate. The determination of viscosity was performed in 20 points with a reading duration of 10 sec and shear rate ranging from 1 to 2001/s. The viscosity measurements were performed according to shear rate and stress applied to the samples. The numerical values of the velocity gradient *D*_*r*_, the modulus of shearing, and flow behavior kinetics were calculated using the Oswald de Waele equation [6].

### 2.10. Thermogravimetric Analysis (TGA) of EPS

Thermogravimetric analysis of the polysaccharide was done using Perkin Elmer, Diamond TGA apparatus using 27.1 mg of the test material. The TGA curve plots the reference material temperature on the *x*-axis against the TGA signal, converted to percentage weight change on the *y*-axis. The EPS was placed in a platinum crucible and heated at a linear rate of 20°C/min over a temperature range of 40°C to 730°C under nitrogen, and the corresponding weight loss was determined.

### 2.11. Scanning Electron Microscopy and Energy Dispersive X-Ray (SEM-EDX) Analysis of EPS

The microstructure and surface morphology of the purified EPS were observed using a Carl Zeiss Supra 55 Gemini (German Technology Jena, Germany) Scanning Electron Microscope at an accelerating voltage of 20 keV. The EPS was mounted on the metal stub and sputtered with gold. Micrographs were recorded at higher magnification to ensure clear images. Elemental composition of the EPS was determined by Energy Dispersive X-Ray analyzer, Oxford Instruments (EDX) equipped with SEM. The X-ray spectrum of the elements was obtained at an accelerating voltage of 15 keV.

### 2.12. Atomic Force Microscopy (AFM) of EPS

Glass slides were treated with a mixture of 15 mL of hydrochloric acid (HCl) and 5 mL of nitric acid (HNO_3_) for 30 min. Subsequently, they were treated with a mixture of 20 mL of sulfuric acid (H_2_SO_4_) and 5 mL of hydrogen peroxide (H_2_O_2_) for 30 min. After rinsing the slides with double distilled water, they were stored in the same until use. Fresh EPS solution was prepared by dissolving in double distilled water. About 10 *μ*L of EPS sample was dropped on the surface of the glass slide and allowed to dry at room temperature. Later the AFM images were obtained by NT-DMT Solver Pro M AFM in semicontact mode using a cantilever with force constant of 5.5–22.5 N/m. The free-standing film of Au and Ag films were stuck to a glass surface. The gold coated silicon probes have the following dimensions: chip size, 3.6*∗*1.6*∗*0.4 mm, the radius of curvature, 10 nm; tip height 10–15 *μ*m. The images were analyzed by using Nova software.

### 2.13. Determination of Total Antioxidant Capacity of EPS

Total antioxidant capacity was prepared by dissolving 1.235 g of ammonium molybdate (4 mM) and 0.9942 g of sodium sulfate (28 mM) and 45 mL of sulfuric acid (0.6 M) into distilled water (250 mL). Various concentrations of EPS (300–1500 *μ*g) were dissolved in 1 mL of total antioxidant capacity. The absorbance was measured at 695 nm after 15 min incubation. Ascorbic acid was used as standard [7].

### 2.14. Determination of Reducing Power of EPS

The reducing power of the EPS was determined according to the method of Oyaizu [8]. Various concentrations of EPS (300–1500 *μ*g) in 1 mL of distilled water were mixed with 2.5 mL of 0.2 M phosphate buffer (pH 6.6) and 2.5 mL of 1% potassium ferricyanide. The mixture was incubated at 50°C for 20 min. Trichloroacetic acid (10%; 2.5 mL) was added to the mixture and centrifuged for 10 min at 3000 rpm. Distilled water (2.5 mL) and 1% ferric chloride (0.5 mL) were added to 2.5 mL of the upper layer. After an incubation of 10 min, the absorbance was read at 700 nm in a spectrophotometer. A higher absorbance indicates the greater reducing power.

### 2.15. Hydrogen Peroxide Scavenging Assay of EPS

The ability of EPS to scavenge hydrogen peroxide was determined according to the method of Ruch et al. [9]. A solution of hydrogen peroxide (10 mM) was prepared in 0.1 M phosphate buffer saline (pH 7.4). Various concentrations (200–1000 *μ*g) of EPS were rapidly mixed with 2 mL of hydrogen peroxide solution. After an incubation of 10 min, the absorbance of hydrogen peroxide was measured at 230 nm. Gallic acid was used as the standard. The percentage scavenging of hydrogen peroxide was calculated using the formula

(1) $$\begin{array}{c} Percentage\;of\;scavenging\;=\frac{\left(A_{0}-A_{1}\right)}{A_{0}}\times 100, \end{array}$$

where *A*_0_ is the absorbance of control and *A*_1_ is the absorbance of sample or standard.

### 2.16. Determination of Emulsification Activity of EPS

The emulsification activity of EPS was done as per Cameron et al. [10] with slight modifications. Various hydrocarbons and vegetable oils were used. Hydrocarbon or vegetable oil (1.5 mL) was added to 1.5 mL of various concentrations of EPS (0.5, 1.0, and 1.5 mg/mL) and vortexed vigorously for 2 min. The emulsification activity (% EA) was determined after 1 hr whereas the emulsion stability was determined as emulsification index (% EI) after 24, 48, and 72 hrs. The % EA and % EI were calculated by dividing the height of the emulsion layer (in cms) by the total height of the mixture (in cms) multiplied by 100.

## 3. Results and Discussion

### 3.1. Identification of EPS Producer

**Figure 1:**
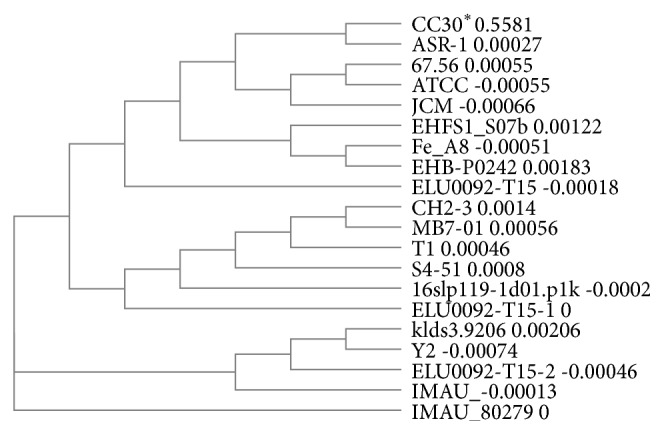
Phylogenetic relationships among different* Streptococcus* species along with strain CC30. ^*∗*^GenBank accession numbers of sequences used: CC30: KY789476; ASR-1: JX275810.1; 67.56: AY687383.1; ATCC: AY687382.1; JCM: LC096241.1; EHFS1_S07b: EU071490.1; Fe_A8: EU735738.1; EHB-P0242: KU978228.1; ELU0092-T15: HQ781886.1; CH2-3: JX079485.1; MB7-01: JQ693082.1; T1: EF990662.1; S4-51: GQ898717.1; 16slp119-1d01.p1k: GQ159653.1; ELU0092-T15-1: HQ781792.1; Klds3.9206: EU660206.1; Y2: DQ911624.1; ELU0092-T15-2: HQ781888.1; IMAU: HM059002.1; IMAU_80279: HM058561.1.

The lactic acid bacteria were identified by the sequencing and phylogenetic analysis of the 16S rRNA gene. As analyzed by BLAST, the sequence showed high (99%) identity with different strains of* Streptococcus thermophilus* (Figure 1). Phylogenetic analysis also placed the strain among* Streptococcus thermophilus.* The phylogenetic tree produced revealed that* S. thermophilus* strains could be clustered into three distinct branches, of which two branches are split into two subbranches. Strain CC30 formed as one of the subbranches along with the other subbranch of ASR-1 strain. CC30 was formed as one cluster along with ASR-1, 67.56, ATCC, JCM, EHFS1_S07b, Fe_A8, EHB-P0242, ELU0092-T15, CH2-3, MB7, T1, S4-51, 16slp119-1d01.p1k, and ELU0092-T15-1. Within this cluster, CC30 exhibited 100% sequence identity with ELU0092-T15, 16slp119-1d01.p1k, and ELU0092-T15-1, proving that these four strains are closely related. The sequence identity with the rest of the strains in the cluster was 99%.

### 3.2. Production and Isolation of EPS

The strain CC30 grown in 11% skimmed milk produced a viscous and ropy curd. EPS was extracted from the ropy curd and dialyzed. The purified fractions of the EPS after ethanol precipitation produced 1.95 g/L of EPS and showed the negligible amount of protein and nucleic acid. Purified EPS was then subjected to morphological, structural, and physicochemical characterization to explore its potential applications.

### 3.3. Fourier Transform Infrared (FTIR) Analysis of EPS

**Figure 2:**
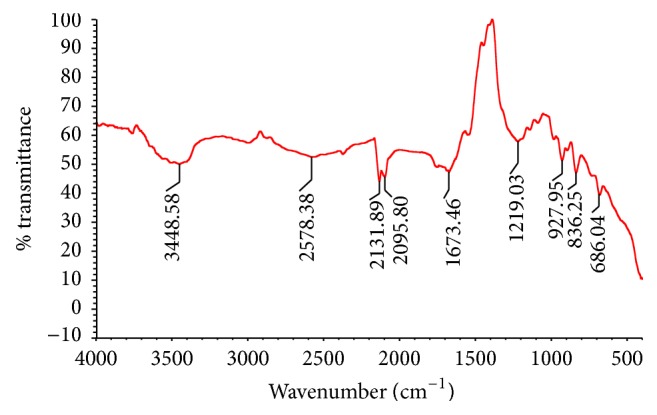
FTIR spectrum of purified EPS of CC30 in the range of 500–4000 cm^−1^.

FTIR is an effective technique that works on the principle that group of bonds vibrates at characteristic frequencies. It can be employed to detect functional groups and for characterizing covalent bonding.  Figure 2 shows the FTIR spectrum of the CC30. A broad stretching at 3448 cm^−1^ represents the stretching vibration of hydroxyl groups and is characteristic of a carbohydrate ring [11, 12]. The absorption band at 2578 cm^−1^ can be assigned to the C-H stretching of methyl or methylene groups, usually present in hexoses like glucose or galactose, or deoxyhexoses like rhamnose or fucose [13]. Two peaks at 2131 cm^−1^ and 2095 cm^−1^ correspond to the presence of free carboxylic groups [14]. In the region 1673 cm^−1^, the absorption band usually represents the stretching vibration of C=O group [15, 16]. Similarly, the peak at 1219 cm^−1^ could be assigned to C-O stretching in ether or alcohol groups [17]. The main absorption band at 927 cm^−1^ indicates the vibrations of the glycoside link C-O-C [18, 19]. In the fingerprint region (the region below 1500 cm^−1^), small peaks indicate the presence of sulfated groups and/or that the substance is polysaccharide [20, 21]. The band at 836 cm^−1^ is characteristic of *α*-D glucan. The absence of characteristic absorption peak around the region of 1700–1770 cm^−1^ suggests that neither glucuronic acid nor diacetyl ester is present in the EPS [21]. The presence of carboxyl groups in the FTIR spectra of the polymer indicates that they may serve as the binding site for divalent cations [22].

### 3.4. Molecular Weight Estimation of EPS

**Figure 3:**
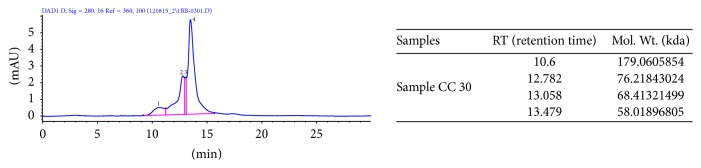
SE-HPLC chromatogram of CC 30 EPS.

Size exclusion or gel permeation chromatography is a chromatographic technique in which separation of analytes is done based on their molecular size. Four fractions of different molecular weights were obtained in the range 58 kDa to 180 kDa (Figure 3). Other reports also mention the presence of different fractions in the EPS of LAB [23–27].

The molecular mass of heteropolysaccharides may range from 4.0 *∗* 10^4^ and 6.0 *∗* 10^6^ [27];* Streptococcus thermophilus* SY2 produced two EPS fraction of 2 *∗* 10^6^ and 5 *∗* 10^4^ [26].* Lactobacillus paracasei* produces two eps fractions of 4 *∗* 10^4^ Da and 1.36 *∗* 10^4^ Da [25].* Lactobacillus plantarum* was reported to have a molecular weight of 2.68 *∗* 10^5^ Da [13], while* Streptococcus thermophilus *produces EPS with the molecular weight of 1 *∗* 10^6^ Da [28]. Other strains of* Streptococcus thermophilus* have been reported to produce high molecular weight EPS with glucose and galactose as the monosaccharide units [1, 29, 30].

### 3.5. Monosaccharide Composition Analysis of EPS

**Figure 4:**
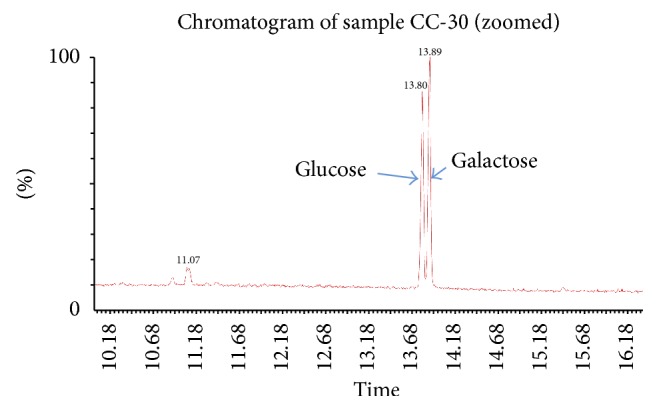
GC-MS chromatogram of CC30 EPS.

Analysis of the exopolysaccharide to determine the sugar composition was done by using Gas Chromatography-Mass Spectroscopy (GCMS; Figure 4). CC30 EPS is composed of glucose and galactose in the molar ratio of 1.4 : 1.6. The sugars, glucose, and galactose are present almost in the same proportion. This result is in line with previous reports of EPS from* Streptococcus thermophilus.* Strains SFi39, SY89, and SY102 contain both glucose and galactose and are often found in the same proportion with the molar ratios of 1 : 1 [29, 31]. Qin et al. [30] and Zhang et al. [1] in their study reported that EPS produced by* Streptococcus thermophilus 05-34 *and ST1 contained only galactose and glucose with varying molar ratio (1 : 0.8, 1 : 2, resp.). The occurrence of different saccharide moieties suggests that the EPS produced is a heteropolysaccharide.* Streptococcus thermophilus* has been reported to produce heteropolysaccharide [28, 29, 32, 33].

### 3.6. Rheological Analysis of Aqueous EPS Solution

The EPS produced by LAB are of a large technological interest since they improve the rheological characteristics of the products and help reduce syneresis [18]. The rheological properties of the exopolysaccharide were investigated to determine its potential application as a thickener or gelatinizer in the food industry. The dynamic viscosity of EPS was measured, and its flow behavior kinetics was analyzed. The power law model for a non-Newtonian flow is known as Oswald de Waele equation [6].

(2) $$\begin{array}{c} \tau ={KD_{r}}^{n}, \end{array}$$

where *τ* is shear stress (Pa), *D*_*r*_ is shear rate (S^−1^), *K* is consistency index (Pa S^*n*^), and *n* is flow index value.

**Figure 5:**
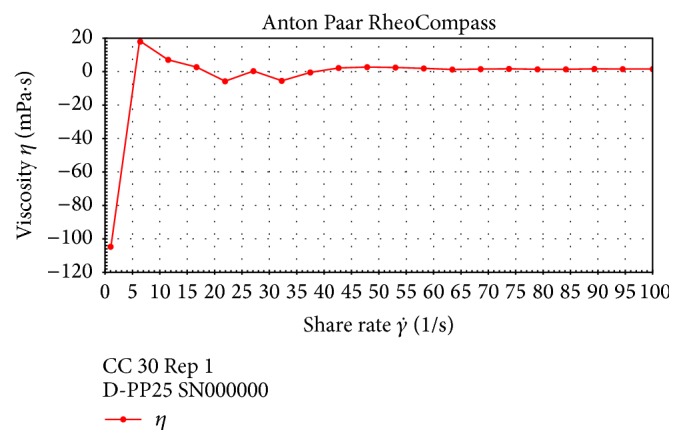
Rheological behavior of CC30 EPS in distilled water.

The flow index value *n* of the EPS was −0.587, which indicates the pseudoplastic nature of the EPS (Figure 5). Increasing the shear rate from 0 to 100 s^−1^ resulted, initially, in increased viscosity of the sample followed by a decrease and stabilization.

Aqueous solutions of polysaccharides are characterized by the decrease in the apparent viscosity of the solution with increasing shear stress. Hence, EPS from CC30 can be used as a thickening and gelling agent.

### 3.7. Thermogravimetric Analysis of EPS

The thermogravimetric analysis of EPS from CC30 was carried out dynamically between temperature and weight loss. According to Fagerson [34], when the EPS was subjected to different temperatures, the primary events that occur with the initial increase in temperature are gelatinization and swelling. Further increase in temperature causes dehydration and pyrolysis of the exopolysaccharide.

**Figure 6:**
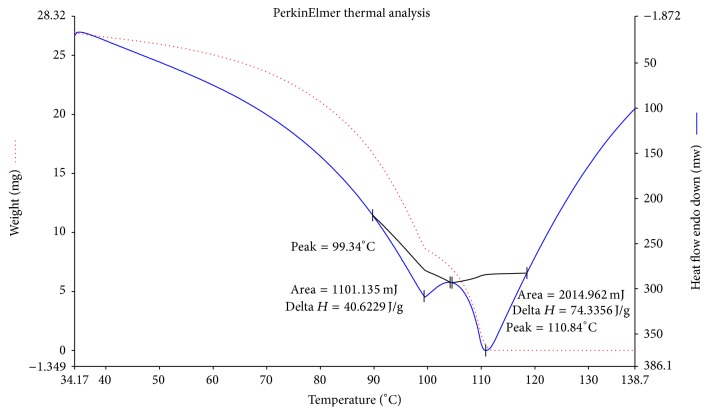
Thermogravimetric mass loss spectrum of EPS from CC30.

As shown in Figure 6, the EPS showed an initial weight loss between 50 and 99°C. This initial weight loss may be associated with the loss of moisture. Many reports suggest that initial loss of moisture is due to the carboxyl groups which are present in high level and are bound to water molecules [21]. Thus, the initial weight loss by CC30 EPS is due to the presence of high content of carboxyl groups. At a degradation temperature (*T*_*d*_) of 110.84°C, a large weight loss could be observed. This fact implies that the EPS of CC30 should not be submitted to temperature ranges close to 100°C [35]. The second phase (dotted line) shows a release of energy that is representation of an exothermic process with a maximum of 355°C, where large mass loss can be observed. The difference between the *T*_*d*_ values of different EPSs is attributed to their different structural composition. An important characteristic considered for industrial application of EPS is thermal stability, especially in the food industry, since most of the manufacturing and processing of the food products are carried out usually at high temperatures [36]. The EPS of CC30 being thermostable and with its rheological properties can be considered as an ideal candidate for food application.

### 3.8. Scanning Electron Microscopy and Energy Dispersive X-Ray Analysis of EPS

**Figure 7:**
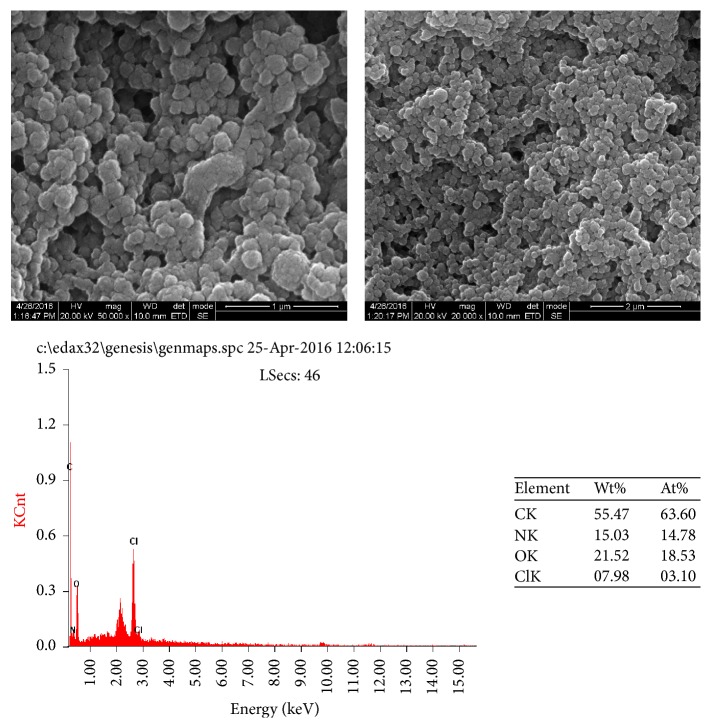
SEM images and EDX spectrum of purified EPS of CC30. Wt%: percentage by weight; At%: percentage by atomic weight. CK: carbon; NK: nitrogen; OK: oxygen; ClK: chlorine.

The scanning electron micrographs and elemental composition by the Energy Dispersive X-Ray analysis of EPS are represented in Figure 7. The EPS showed a three-dimensional structure with irregular lumps of different size and with a coarse surface. Upon higher magnification, the spherical structure of the EPS can be observed. The EPS possess porous nature. The EPS from* Lactobacillus plantarum* was observed to have the porous web-like structure [21]. SEM micrographs of EPS from* Lactobacillus fermentum* CFR2195 showed flake-like structural units and were highly compact [15]. The different morphology and topography of different polysaccharides were most likely caused by the difference in sample extraction, preparation, and purification and by the difference in the physicochemical properties of the exopolysaccharide. The elemental composition of the CC30 EPS showed the composition of carbon, oxygen, nitrogen, and chlorine. Based upon our literature search, there were no reports showing the surface morphology and elemental composition of the EPS produced by* Streptococcus thermophilus.*

### 3.9. Atomic Force Microscopy of EPS

**Figure 8:**
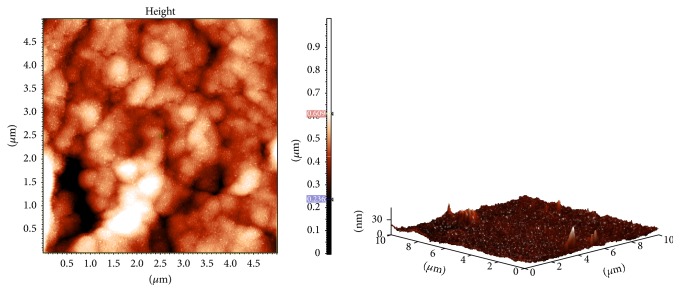
Atomic Force Microscopy (AFM) images of CC30 EPS.

The three-dimensional surface structure and roughness of the EPS can be analyzed by Atomic Force Microscopy (AFM) which has been used extensively in the recent times to characterize the morphological features, the conformation of the individual macromolecules, and the dynamics of the polymer. The topographical AFM images of the CC30 EPS are shown in Figure 8. CC30 EPS deposited from aqueous solution have spike-like lumps with height ranged from 10 to 30 nm. Since the molecules are tightly packed, it can be suggested that the CC30 EPS has strong affinity for water molecules and has a pseudoplastic behavior [21, 37]. The result was in line with those reported by Sajna et al. [36] for* Pseudozyma* EPS. Different shapes and structures have been reported for different EPS. Li et al. [38] showed that the EPS of* Lactobacillus helveticus *has rounded to spherical lumps of different sizes. Ahmed et al. [37] found the reticulate shape for the ZW3 EPS. Other strains of* Streptococcus thermophilus* 05-34 showed different topography but the glucose and galactose as monosaccharide units. The EPS shows the presence of irregular lumps and short chains of 10 to 100 nm in length [30]. There were no other studies reporting AFM study of EPS produced by* Streptococcus thermophilus*. The viscous nature of the CC30 EPS might be caused by its water binding nature.

### 3.10. Determination of Total Antioxidant Capacity of EPS

**Figure 9:**
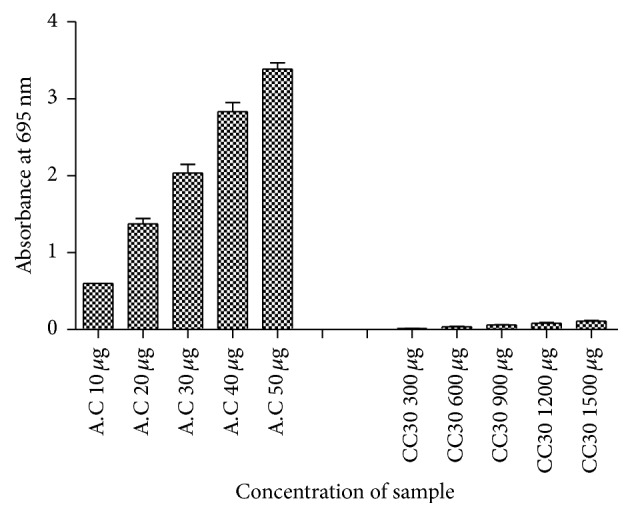
Total antioxidant capacity of EPS (CC30) compared with standard ascorbic acid (AC).

The antioxidant activity of the CC30 EPS is shown in Figure 9. The antioxidant potential of EPS exhibited a dose dependent activity within a concentration range of 3–1.5 mg/mL. However, the antioxidant capacity was lower than that of ascorbic acid, which indicated that the EPS has moderate antioxidant capacity.

### 3.11. Determination of Reducing Power of EPS

**Figure 10:**
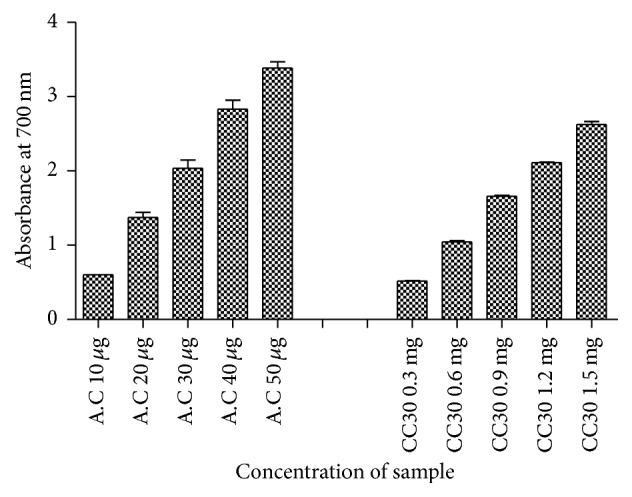
Reducing power of the EPS extracts.

Figure 10 shows the reducing power of EPS compared to ascorbic acid. Reductive ability was measured by investigating the Fe^3+^-Fe^2+^ transformation in the presence of EPS samples. Like the antioxidant capacity, the reducing activity of EPS exhibited a dose dependent activity. As the concentration increased, the reducing power of the EPS extracts also increased. All concentrations of the EPS showed higher activities than the control.

### 3.12. Hydrogen Peroxide Scavenging Assay of EPS

**Figure 11:**
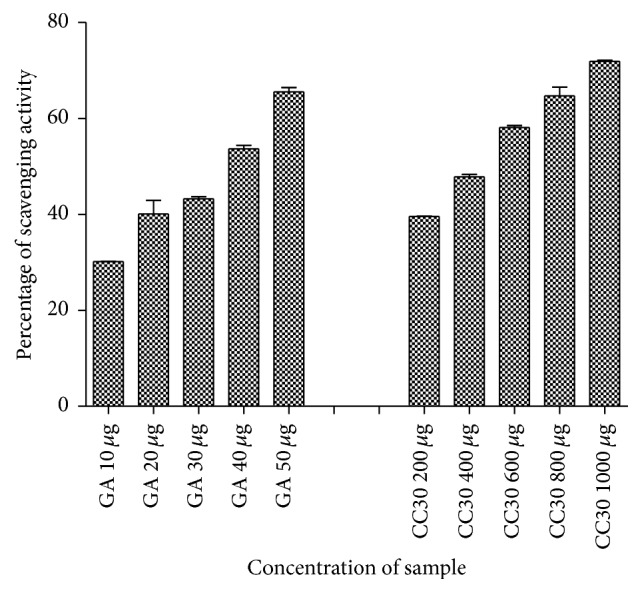
Hydrogen peroxide scavenging activities of EPS extracts compared with standard gallic acid (GA).

Hydrogen peroxide has strong oxidizing properties. The ability of various concentrations of EPS of CC30 to scavenge hydrogen peroxide as compared with that of standard Gallic acid is shown in Figure 11. From the results we can infer that the highest scavenging activity of the EPS was observed at 1000 *μ*g/mL concentration (71.9 ± 0.2%). The EPS of CC30 has significant hydrogen peroxide scavenging activity.

### 3.13. Determination of Emulsification Activity (EA)

Emulsification indices (EI) of the EPS with different solvents are shown in Table 1. An emulsion was prepared with different vegetable oils (coconut oil, palm oil, and sunflower oil) and hydrocarbons (petrol, diesel, kerosene, and xylene). The EPS of CC30 was capable of stabilizing emulsions with different oils and hydrocarbons. The index values were higher for sunflower oil (58%). Both EA and EI for sunflower oil were higher when compared with those of other solvents. CC30 EPS can be used as emulsifier in foods, especially, where sunflower oil is used. EPS produced by* A. pullulans* showed maximum stability with olive oil (56%) [39] whereas the EPS produced by* Bacillus megaterium *showed the highest emulsion index with coconut oil (76%) [40].

## 4. Conclusion

Being an EPS producer,* Streptococcus thermophilus* CC30 may be employed in the production of fermented foods like yoghurt. The EPS produced has molecular weight in the range of 58 to 180 KDa with glucose and galactose as monomers. Its thermal stability along with rheological property can be exploited in food industry. Its reducing activity and hydrogen peroxide radical scavenging activity and moderate antioxidant activity indicate pharmacological applications and inclusion in functional foods. The purified EPS of CC30 can be a potential emulsifier.

## Figures and Tables

**Table 1:** Emulsifying activity (EA)^*∗*^ of extracellular polysaccharide with different concentrations under different vegetable oils and hydrocarbons.

Hydrocarbon/oil	EPS mg/mL	% EA	Emulsifying index^*∗∗*^		
			*E*_24_	*E*_48_	*E*_72_
Petrol	0.5	54.95 ± 2.3	61.1 ± 2.6	65.48 ± 3.5	65.48 ± 3.5
	1	54.95 ± 2.4	56.89 ± 2.4	69.33 ± 3.7	64.17 ± 1.7
	1.5	54.95 ± 2.5	61.1 ± 2.6	60.71 ± 5.0	65.47 ± 1.6

Diesel	0.5	48.48 ± 4.2	54.68 ± 2.2	54.54 ± 0	59.43 ± 2.6
	1	48.48 ± 4.3	53.02 ± 2.1	54.54 ± 1	56.3 ± 2.4
	1.5	62.11 ± 2.1	54.68 ± 2.2	49.24 ± 1.0	46.91 ± 2.0

Kerosene	0.5	53.12 ± 4.4	54.83 ± 0	53.12 ± 0	53.97 ± 1.2
	1	53.12 ± 4.4	51.61 ± 0	53.12 ± 0	54.83 ± 0
	1.5	53.12 ± 4.4	48.48 ± 0	52.41 ± 3.4	51.47 ± 2.0

Coconut oil	0.5	21.66 ± 2.3	31.58 ± 0.7	34.99 ± 2.3	34.99 ± 2.3
	1	24.24 ± 0	40.6 ± 0	37.87 ± 6.4	43.08 ± 0.9
	1.5	31.81 ± 2.1	56.3 ± 2.4	50.9 ± 3.4	46.96 ± 2.1

Palm oil	0.5	51.66 ± 2.3	55.17 ± 0	53.39 ± 2.5	56.15 ± 1.3
	1	51.66 ± 2.3	55.17 ± 0	49.99 ± 4.7	49.99 ± 4.7
	1.5	54.99 ± 2.3	52.47 ± 1.2	56.72 ± 2.6	56.72 ± 2.6

Sunflower oil	0.5	58.16 ± 0.1	56.66 ± 0	53.33 ± 4.7	53.33 ± 4.7
	1	58.06 ± 0	56.66 ± 0	56.3 ± 2.4	56.3 ± 2.4
	1.5	50.80 ± 1.1	53.12 ± 0	51.56 ± 2.2	51.56 ± 2.2

Xylene	0.5	56.44 ± 2.2	56.44 ± 2.2	47.72 ± 3.2	46.91 ± 2.0
	1	62.35 ± 0.2	53.22 ± 2.2	50.8 ± 1.1	50.08 ± 1.1
	1.5	47.62 ± 1.0	49.99 ± 2.2	51.60 ± 4.5	49.19 ± 1.1

^*∗*^Values are mean ± standard deviation (*n* = 2). ^*∗∗*^Subscript indicates hours of incubation with solvent/oil.
